# Supplementary material for: The genome sequence of a soldier fly, Nemotelus pantherinus (Linnaeus, 1758)
Source: Wellcome Open Res. 2025 Jun 2;10:303. [Version 1] doi: 10.12688/wellcomeopenres.24309.1 (PMC12181765; doi:10.12688/wellcomeopenres.24309.1)
Supplement: Peer review history

# Reviewer report: Reviewer response for version 1

**DOI:** 10.21956/wellcomeopenres.26809.r125031

**Published:** 2025-06-20

**Authors:** Komal Kumar Raja

**Affiliations:** 1 Baylor College of Medicine, Houston, Texas, USA

The article by Irwin A., Mitchell R., et al. presents the genome sequence of *Nemotelus pantherinus* with a total length of 804.6 megabases. The authors have identified 27,389 protein-coding genes in the genome. The authors briefly introduce the morphological and ecological aspects of this species. The Hi-C map shows evidence of good quality with strong diagonal signal. The number of scaffolds is low with the biggest scaffold being 270M, which is excellent. The BUSCO scores suggest strong completeness and minimal fragmentation. The methodology is good as well.

 Overall, this is a **high-quality, highly contiguous genome assembly and is a good genomic resource.**

Are sufficient details of methods and materials provided to allow replication by others?

Yes

Is the rationale for creating the dataset(s) clearly described?

Yes

Are the datasets clearly presented in a useable and accessible format?

Yes

Are the protocols appropriate and is the work technically sound?

Yes

Reviewer Expertise:

Genomics, single cell genomics, Bioinformatics and Neurobiology

I confirm that I have read this submission and believe that I have an appropriate level of expertise to confirm that it is of an acceptable scientific standard.

# Reviewer report: Reviewer response for version 1

**DOI:** 10.21956/wellcomeopenres.26809.r125028

**Published:** 2025-06-17

**Authors:** Henrique Antoniolli

**Affiliations:** 1 Universidade Federal do Rio Grande do Sul (UFRGS), Porto Alegre, Brazil

The present study reports a chromosome-level assembly and the mitochondrial genome for a soldier fly *Nemotelus pantherinus*. The background section brings an overview on its general biology, along with the rationale for sequencing its genome. Methods are well described and the employed software is properly cited. The assembly achieved satisfactory levels of completeness.

 Minor issue:

- The mitochondrial genome should also have its genes predicted and annotated.

Are sufficient details of methods and materials provided to allow replication by others?

Yes

Is the rationale for creating the dataset(s) clearly described?

Yes

Are the datasets clearly presented in a useable and accessible format?

Yes

Are the protocols appropriate and is the work technically sound?

Yes

Reviewer Expertise:

Genomics, evolution

I confirm that I have read this submission and believe that I have an appropriate level of expertise to confirm that it is of an acceptable scientific standard.
